# Video to Home Delivery of Evidence-Based Psychotherapy to Veterans With Posttraumatic Stress Disorder

**DOI:** 10.3389/fpsyt.2019.00893

**Published:** 2019-12-06

**Authors:** Derrecka M. Boykin, Fallon Keegan, Karin E. Thompson, Emily Voelkel, Jan A. Lindsay, Terri L. Fletcher

**Affiliations:** ^1^VA South Central Mental Illness Research, Education and Clinical Center, Houston, TX, United States; ^2^Houston VA HSR&D Center for Innovations in Quality, Effectiveness, and Safety, Michael E. DeBakey VA Medical Center, Houston, TX, United States; ^3^Menninger Department of Psychiatry and Behavioral Sciences, Baylor College of Medicine, Houston, TX, United States; ^4^Department of Psychology, University of North Texas, Denton, TX, United States; ^5^Michael E. DeBakey VA Medical Center, Houston, TX, United States

**Keywords:** telehealth, telemental health, posttraumatic stress disorder, evidence-based psychotherapy, veterans

## Abstract

**Background:** The Veterans Health Administration (VHA) has pioneered the implementation of video to home (VTH) technology to increase access to mental health treatments for Veterans facing barriers to receiving in-person care, particularly for posttraumatic stress disorder (PTSD). Randomized controlled trials have established the noninferiority of evidence-based psychotherapies (EBPs) for PTSD delivered through VTH, compared to in-person delivery. Less is known about the use of VTH to deliver EBPs for PTSD in routine clinical practice.

**Objective:** We examined the provision of EBPs for PTSD delivered *via* VTH at a large Southwestern VHA PTSD outpatient clinic.

**Methods:** Data were obtained from chart review of the electronic medical records of Veterans receiving at least one session of Cognitive Processing Therapy or Prolonged Exposure *via* VTH in the VHA PTSD clinic during the study time frame.

**Results:** Fourteen providers (including six psychology trainees) delivered EBPs for PTSD *via* VTH between 2016 and 2018. Providers treated 74 Veterans (33.8% women) from diverse sociocultural backgrounds who ranged in age from 25 to 79. Each provider treated about 3.08 (± 2.18) Veterans using VTH, not including one provider who saw more than 30. A hybrid approach, in which VTH-delivery was coupled with in-person delivery, was used with 70.3% of Veterans across treatment (including sessions completed before initiation and after termination of the EBP). This demonstrates the versatility of VTH for meeting individual patient needs. Most EBP sessions (85.4%) were conducted over VTH. Despite Veterans attending an average of 6.85 (± 4.88) EBP sessions, 50% terminated before session 7. This dropout rate is consistent with national and local EBP completion averages within the VHA. Veterans receiving Cognitive Processing Therapy *via* VTH were more likely to complete treatment than those receiving Prolonged Exposure. No other patient factors predicted attrition.

**Conclusions:** This study highlights the use of VTH as “tool in the toolbox” that expands the scope of practice for providers and increases opportunities for Veterans to receive EBPs for PTSD. We describe other potential advantages of using VTH to deliver EBPs for PTSD.

## Introduction

Evidence-based psychotherapies (EBPs), including Cognitive Processing Therapy and Prolonged Exposure, are considered first-line interventions for posttraumatic stress disorder (PTSD) and have strong support in Veteran samples (1–3). Despite the resources the Veterans Health Administration (VHA) has invested in these two therapies, Veteran access to and engagement in EBPs for PTSD remain a challenge (4). Relatively few Veterans with PTSD (9%) receive these EBPs (5–7), and approximately half who initiate these treatments terminate prematurely and do not receive a full course of therapy (8, 9). Notable barriers to accessing and engaging in EBPs include logistic issues such as taking time off work or school, arranging child or elder care, physical limitations, and transportation and travel costs (10, 11). Additional barriers include stigma around seeking mental health care (12) and clinical symptoms such as anxiety (13) that may interfere in seeking PTSD care at the VHA. Some women Veterans and Veterans who experienced military sexual trauma (MST) also express discomfort in VHA clinics (14, 15).

The provision of EBPs *via* video to home (VTH) technology is one promising option for overcoming barriers to care and increasing access to and retention in EBPs for PTSD. VTH is a safe and convenient alternative to in-person care and offers patients the opportunity to receive PTSD treatment in the comfort of their home, work, or other private location. Delivery of EBPs *via* VTH allows patients and providers to synchronously connect for live, interactive therapy sessions using a secure web-based videoconferencing feature on a personal computer, laptop, tablet, or other device. The content of the VTH-delivered EBPs for PTSD is identical to traditional in-person delivery.

Patients report high satisfaction with VTH-delivered care (16), and noninferiority trials demonstrate the clinical effectiveness of EBPs for PTSD delivered *via* VTH compared to in-person treatment, though sample sizes were relatively small (17, 18). Although these randomized controlled trials found no differences in dropout between VTH and in-person treatment, this may be because patients were randomized to a condition and were not able to select which delivery option they preferred. Nonetheless, among patients who terminated therapy prematurely, patients receiving care *via* VTH engaged in more sessions prior to dropout than patients who received in-person care (19).

Although these studies demonstrate the effectiveness of VTH-delivered PTSD treatment and suggest promise in increasing treatment retention, little is known about the current use of VTH to deliver EBPs for PTSD in routine clinical practice. A recent study found that, although VTH use is increasing across the VHA, growth is slow; and the number of Veterans receiving mental health care through VTH is still small (13). One successful model for implementing VTH for EBPs is to integrate VTH across multiple providers in a clinic, rather than designating certain providers (often in remote telehealth centers) to provide VTH. This integrated model allows patients and providers to collaboratively decide whether to conduct all EBP sessions *via* VTH, or whether a hybrid approach of both VTH and in-person sessions is preferable. This Personalized Implementation of Video Telehealth approach has demonstrated success in increasing both provider and patient adoption of VTH for mental health treatment (13, 20).

The goal of this study was to examine the provision of EBPs for PTSD delivered *via* VTH within an outpatient PTSD clinic at the Houston Veterans Affairs Medical Center that uses an integrated model of VTH implementation. Specific aims were to: 1) describe demographic and clinical characteristics of Veterans receiving VTH-delivered EBPs for PTSD, 2) report the number of PTSD clinic providers delivering these EBPs *via* VTH during the study period, 3) report the average number of Veterans each provider treated *via* VTH, 4) report the percentage of Veterans who engaged in the EBP for PTSD exclusively *via* VTH vs. both VTH and in-person, and 5) report rates of VTH EBP for PTSD completion. We also examined known predictors of in-person EBP for PTSD completion including demographic characteristics (e.g., gender, race/ethnic minority), symptom severity, comorbid substance use disorder, service-connection for a disability, and military service era (6, 21, 22) to determine whether these variables predicted VTH EBP for PTSD completion. Given noted differences in completion of Cognitive Processing Therapy compared to Prolonged Exposure, we also included EBP treatment type as a potential predictor (8).

## Methods

### Treatment Setting

The VHA PTSD outpatient clinic is a subspecialty clinic located within the General Mental Health Clinic. The VHA PTSD Clinic at the Houston Veterans Affairs Medical Center had 9 full-time VHA providers and 1 half-time provider (7.5 psychologists and 2 social workers) and employed two to three psychology interns and fellows at a time (their cases are included here). VHA PTSD clinic staff and trainees treat Veterans with a primary diagnosis of PTSD or Other Trauma/Stressor-Related Disorder. Of note, a diagnosis of military-related PTSD is not required for enrollment and Veterans are not excluded for having comorbid psychiatric diagnoses. The VHA PTSD clinic routinely offers Cognitive Processing Therapy and Prolonged Exposure as first-line, gold standard EBPs for PTSD. The VHA PTSD clinic staff were introduced to and gradually trained in VTH starting in 2015 as part of on-going implementation efforts to increase adoption of this clinical innovation (13).

### Data Collection

We reviewed the electronic medical records of all Veterans enrolled in the VHA PTSD clinic who completed at least one individual EBP for PTSD session *via* VTH between January 1, 2016, and December 31, 2018. The study protocol was reviewed and approved by the Institutional Review Board at Baylor College of Medicine and Affiliated Hospitals in addition to the Research and Development committee at the Houston Veterans Affairs Medical Center. Because this study involved a retrospective review of patient medical records, a waiver of informed consent/HIPAA authorization was required and granted. De-identified data (described below) were collected in a SPSS database securely stored behind a VHA firewall only accessible by the principal investigator and approved research staff. Identifiable information was only used to collect aggregate data from the medical records and no identifiable data was downloaded, copied, or printed.

#### Veteran Demographic and Clinical Characteristics

Demographic data were age (in years), gender, race, ethnicity, marital status, academic status (i.e., college student vs. nonstudent), employment, rural or urban residence, military service era, deployment status, and VHA service-connected disability status for PTSD. Veterans' residency zip codes were classified as rural or urban according to the Rural-Urban Commuting Area Codes (23). Information regarding comorbid medical and psychiatric diagnoses was taken from Veterans' problem lists. Medical diagnoses were weighted and summed to compute the Charlson Comorbidity Index, an indicator of disease burden and mortality risk (24). We also tracked whether Veterans had been prescribed recommended medications for PTSD (25).

#### Treatment History

Veterans' treatment history was gathered from two time intervals—the year preceding admission into the VHA PTSD clinic and during the most recent episode of care. The most recent episode of care was defined as the period during which the Veteran completed at least one EBP for PTSD session *via* VTH. Key variables collected at both time intervals were: use of mental health services within this or other VHA clinics (e.g., primary care), medication management visits, use of emergency services (including crisis line calls), and inpatient hospitalizations. Veterans who received psychotherapy (either group or individual) in the VHA PTSD clinic prior to their most recent episode of care were distinguished from Veterans who were newly admitted or never received treatment after admission into the clinic.

#### EBP Treatment Delivered *via* VTH

We extracted treatment information about the most recent episode of care. Provider information, EBP type (Prolonged Exposure or Cognitive Processing Therapy), and nature of primary or index trauma were recorded. Other treatment variables were number of individual sessions (including sessions completed before initiation or completion of EBP), number of EBP sessions, number of missed and cancelled sessions, and notable VTH connectivity issues (e.g., "session terminated early due to poor audio quality"). We differentiated between sessions delivered in-person vs. those delivered through VTH. A 7-session benchmark was used to define EBP completion based on existing definitions of EBP for PTSD completion (6, 26–28). Initial and last recorded PTSD Checklist for *DSM-5* severity scores (29) were collected. Unfortunately, the last recorded PTSD severity scores were available for 24 of 74 Veterans and, thus, we omitted them from data analysis.

### Data Integrity

Deidentified chart review data were entered into a shared project database by two research assistants, who had experience conducting VHA chart review research and had obtained thorough training for the current study. The first author (D.B.) oversaw chart review and met regularly with the research assistants to resolve any emergent problems. For any issues, the first author reviewed the individual charts, and the group reached a consensus.

### Data Analyses

Descriptive statistics of Veteran characteristics and treatment history, provider characteristics, and clinical use of VTH relative to in-person service delivery of EBPs were reviewed. We used logistic regression analysis to examine predictors of EBP completion. Predictors were Veteran demographic and clinical characteristics (e.g., race/ethnicity, service era, and medical/psychiatric comorbidities), EBP type, index trauma type, and prior treatment history. Due to concerns about adequate power, predictors for the logistic regression analysis were purposefully selected by first conducting a univariate analysis of each predictor to determine which variables should be included in the final multivariate model. Predictors that had a significant univariate test at *p ≤* 0.25 process were included in the final model (30).

## Results

### Veteran Characteristics and Treatment History

A total of 74 Veterans (49 men, 25 women) received an EBP for PTSD *via* VTH within the VHA PTSD clinic during the study time fame. Veteran demographic and clinical characteristics are described in **Table 1**. Veterans ranged in age from 26 to 79 (*M* = 42.01 ± 11.71). Most Veterans were Caucasian or African American, non-Hispanic/Latino, married, employed, receiving VHA service connection for a PTSD disability, and living at an urban residence at the time of admission into the clinic. Most Veterans had been deployed to a combat zone with many serving during the Operation Iraqi Freedom/Operation Enduring Freedom, or Gulf War eras. Almost all Veterans (73 of 74) had co-occurring psychiatric diagnoses, most often depression (52.7%) or a substance/alcohol use disorder (24.3%). Fewer Veterans (23 of 74) had significant medical comorbidities. Overall, this sample had low Charlson Comorbidity Index scores indicating low overall disease burden and mortality risk. Combat (54.1%) was the most commonly identified index trauma, followed by MST (20.3%); other military, noncombat trauma (16.2%; e.g., training accidents); and nonmilitary, civilian trauma (9.5%).

**Table 1:** Characteristics of Veterans with PTSD receiving VTH at VHA PTSD Clinic between 2016 and 2018.

Personal characteristic	*N* (%)	*M ± SD*
Age (in years)		42.01 ± 11.71
Gender		
Male	49 (66.1%)	
Female	25 (33.8%)	
Race^a^		
Caucasian	38 (51.4%)	
African American	34 (45.9%)	
Other/Multiracial	1 (1.4%)	
Ethnicity^a^		
Non-Hispanic or Latino	60 (81.1%)	
Hispanic or Latino	13 (17.6%)	
Marital status		
Never Married/Single	19 (25.7%)	
Married	34 (45.9%)	
Divorced/Separated	18 (24.3%)	
Widowed	3 (4.1%)	
% enrolled in college^b^	15 (20.3%)	
% employed^c^	39 (52.7%)	
% living in urban area	66 (89.2%)	
Service Era		
Vietnam	3 (4.1%)	
Post-Vietnam	8 (10.8%)	
Persian Gulf	16 (21.6%)	
OIF/OEF	47 (63.5%)	
% deployed to combat zone	45 (60.8%)	
% service-connected for PTSD disability	48 (64.9%)	
% with comorbid psychiatric diagnoses	73 (98.6%)	
% with comorbid medical diagnoses	23 (31.1%)	
Charlson comorbidity index		0.51 ± 0.91
PTSD severity at VHA PTSD Clinic Intake		60.09 ± 11.79

N, 74; VHA, Veterans Health Administration; OIF/OEF, Operation Iraqi Freedom/Operation Enduring Freedom; PTSD, Posttraumatic Stress Disorder.

^a^n = 73. ^b^n = 64. ^c^n = 63.

**Table 2** describes Veterans' treatment history as it relates to use of outpatient psychotherapy, medical management, emergency services, and inpatient hospitalization prior to and during individual treatment in the VHA PTSD clinic. Most Veterans had received outpatient psychotherapy (including group therapy) prior to their most recent episode of care in the VHA PTSD clinic. Notably fewer Veterans were participating in concurrent outpatient psychotherapy once they started an EBP for PTSD. The majority of Veterans (82.4%) were taking psychotropic medications for PTSD; however, only a quarter used medication management services during the study time frame. Less than 10% of Veterans sought emergency services (including contacting the crisis line) or were hospitalized for urgent mental health crises.

**Table 2:** Percent of Veterans receiving VHA Services before and during VTH Administration in VHA PTSD Clinic.

VHA service type	Prior to VHA PTSD clinic enrollment	During VTH administration
Outpatient psychotherapy	54 (73.0%)	10 (13.5%)
Medication management visits	21 (28.4%)	25 (33.8%)
Emergency services	6 (8.1%)	3 (4.1%)
Inpatient hospitalization	3 (4.1%)	1 (1.4%)

N, 74; VHA, Veterans Health Administration; PTSD, Posttraumatic Stress Disorder.

### Clinical Utilization of VTH

The number of clinic providers delivering EBPs for PTSD *via* VTH steadily increased from 2016 to 2018. In 2016, four clinic providers (including one trainee) used this treatment modality with at least one patient. Seven additional providers (including four trainees) delivered EBPs *via* VTH in 2017, followed by three more providers (including one trainees) in 2018. Altogether, 14 providers (8 of 9.5 clinical staff and six trainees) administered EBPs for PTSD using VTH during the study period. On average, each provider delivered care to approximately 3.08 (± 2.18) Veterans *via* VTH, not including one provider who saw more than 30 Veterans. Between 24 and 26 Veterans were seen each year during the study time frame.

Including sessions completed before initiation and after termination of an EBP for PTSD, Veterans were seen for 11.08 (±11.19) total sessions. A minority of Veterans (29.7%) completed individual sessions that were delivered exclusively through VTH. Most often, a hybrid approach in which VTH-delivery was coupled with in-person delivery was used. Of those receiving this hybrid approach (*n* = 52), 67.8% of sessions were delivered *via* VTH. Fifty-seven of 74 (77%) Veterans missed or cancelled an average of 2.77 (±2.59) scheduled sessions. Technical difficulties associated with VTH ranged in severity from temporary audio or video loss to major broadband issues (e.g., lost wireless connection). Significant technical difficulties that disrupted the quality of sessions (e.g., persistent loss of audio or video) or led to early termination of a session were experienced once or twice during treatment by 54.0% of the sample.

### EBP Completion During VTH Delivery

Cognitive Processing Therapy was administered more often than Prolonged Exposure (75.7% vs. 24.3%, respectively). Due to the small number of Veterans receiving Prolonged Exposure (*n* = 18), Prolonged Exposure and Cognitive Processing Therapy were examined together for the remaining analyses. Veterans attended an average of 6.85 (±4.88) EBP for PTSD sessions, with 85.4% of these sessions conducted over VTH. Thirty-seven (50%) Veterans completed treatment (i.e., attended seven or more EBP sessions).

Univariate and multivariate logistic regression results are shown in **Table 3**. Predictors of treatment completion that had significant univariate logistic regression results were gender, service-connected PTSD disability, service era, EBP type, index trauma type, prior psychotherapy in the VHA PTSD clinic, and prior outpatient psychotherapy (see **Table 2**). When these variables were placed into a multivariate logistic regression model, only EBP type remained a significant predictor at *p* < .05 (see **Table 2**). This finding suggests that Veterans receiving Cognitive Processing Therapy *via* VTH were more likely to complete treatment than Veterans receiving Prolonged Exposure.

**Table 3:** Logistic regression predicting completion of EBPs for PTSD delivered using VTH.

Predictor	Univariate analyses			Multivariate analysis		
	OR	95% CI	*p*	OR	95% CI	*p*
Age (in years)	1.02	(0.98, 1.06)	0.444			
Gender						
Male	1.00		0.089	1.00		0.618
Female	0.42	(0.16, 1.14)		0.73	(0.21, 2.55)	
Race/Ethnicity						
Non-Hispanic White	1.00		0.809			
Minority	0.89	(0.35, 2.29)				
Service Era						
OIF/OEF Era	1.00		0.229	1.00		0.788
Other Service Eras	1.80	(0.69, 4.69)		1.18	(0.35, 3.97)	
Service-connected PTSD Disability						
Yes	1.00		0.147	1.00		0.267
No	2.06	(0.78, 5.45)		1.94	(0.60, 6.23)	
Comorbid Substance Use Diagnosis						
Yes	1.00		0.282			
No	1.81	(0.61, 5.36)				
Diagnostic Complexity	1.14	(0.09, 1.90)	0.608			
PTSD Severity at VA PTSD Clinic Intake	1.00	(0.96, 1.04)	0.851			
EBP Treatment Type						
CPT	1.00		0.036	1.00		0.048
PE	3.47	(1.09, 11.05)		3.86	(1.01, 14.75)	
Index Trauma Type						
MST	1.00		0.155	1.00		0.157
Other Military/Nonmilitary Trauma	0.42	(0.13, 1.39)		0.31	(0.06, 1.58)	
Prior Psychotherapy in VA PTSD Clinic						
Yes	1.00		0.215	1.00		0.229
No	2.28	(0.62, 8.35)		2.41	(0.56, 10.07)	
Other Prior Outpatient Psychotherapy						
Yes	1.00		0.282			
No	1.81	(0.61, 5.36)				

N, 74; OIF/OEF, Operation Iraqi Freedom/Operation Enduring Freedom; PTSD, Posttraumatic Stress Disorder; EBP, Evidence-based Psychotherapy; CPT, Cognitive Processing Therapy; PE, Prolonged Exposure; MST, Military sexual trauma.

## Discussion

The current study highlights the broad reach of VTH technology in improving access to EBPs for Veterans with PTSD in routine clinical practice. Our findings support a growing emphasis on clinical expertise and competence in delivery of EBPs *via* VTH as opposed to predetermined criteria to identify which patients might benefit from this treatment modality (31). The present sample was fairly heterogeneous in terms of clinical complexity, yet representative of Veterans who seek VHA care (32). In the past, patients presenting with complex, chronic comorbidities might have been deemed ineligible for VTH services due to concerns about clinical appropriateness and safety. Now, telemental health services are considered a topmost strategy for engaging difficult-to-reach populations, such as Veterans with PTSD (33). VTH eliminates many practical barriers (e.g., transportation issues, long distance from facility, inflexible work schedule) and VHA-system-related barriers (e.g., inflexible clinic scheduling, discomfort with physical VHA environment, overcrowding) that impact utilization of EBPs for PTSD. There are also potential advantages to seeing patients in their home environments, where they may feel safer discussing trauma-related issues. It is, thus, important not to limit use of VTH to straightforward clinical cases (e.g., physical limitations or disabilities) and potentially miss critical opportunities to reach patients who might otherwise not receive care.

Patients from diverse demographic and clinical backgrounds consider home-based videoconferencing technology, such as VTH, highly satisfactory and acceptable (31, 34). The current sample included a mixture of younger and older Veterans from a range of racial/ethnic backgrounds, service eras, etc. There was also a high percentage of women (33.7%), a historically underserved group within the VHA (14.1%; 35). These data challenge preconceived notions about which patients may be interested in receiving technology-based services. Therefore, providers who are or will be integrating VTH into their clinical practice should consider routinely offering this service to patients as an option for care delivery.

The Houston Veterans Affairs Medical Center adopted an integrated model of VTH delivery in which most providers (84.2% of clinical staff and six psychology trainees) offered VTH as one delivery option, rather than designating a smaller number of providers who exclusively offered telehealth services (13). The inclusion of trainees as VTH providers further illustrates efforts to integrate VTH as a standard care option available to all patients by all providers within this clinic. Having multiple providers who can offer VTH decreases the burden of trying to meet diverse patient needs with one or two designated VTH providers. Clinical settings with more than one VTH provider are also better equipped to manage whenever patient needs change (e.g., unexpected changes in work schedule or family obligations). This approach also ensures continuity of care by allowing patients to receive in-person and VTH care from a single provider. As providers in this study demonstrated, integrating VTH-delivery with in-person delivery capitalizes on the flexibility of VTH as an as-needed treatment delivery option that truly promotes a patient-centered model of care that optimizes patient choice (31).

The Houston VHA PTSD clinic, as part of a multisite implementation project (13, 33), received external guidance and support that, in collaboration with the clinic director as a local advocate, facilitated provider adoption of VTH. Another influential factor was the occurrence of Hurricane Harvey in 2017 and provider concerns about Veterans experiencing major disruptions in care in the aftermath of this natural disaster. Providers who had not yet used VTH were eager to learn how to integrate this treatment modality into their practice to minimize undue delays in care. One major advantage to working in VHA and other large healthcare systems is that providers have access to trainings, equipment, and other resources that support the uptake of VTH. For providers in other mental health settings, private practice, or rural communities, establishing a telehealth practice may require additional effort and careful attention to federal and state policies about reimbursement models for treatment delivered remotely [see Campbell et al. (36), see also Yellowless et al. (37)].

Attrition in this study (50%) was on par with local and national in-person EBP completion averages for Veterans with PTSD (6, 28, 38). Unfortunately, present data were insufficient for understanding what contributed to these attrition rates. Treatment response varies and, in the absence of treatment outcome data, we cannot conclude whether noncompleters represent early responders or Veterans who were nonresponsive to treatment (26). Attrition could have also been due to other unknown factors, such as patient relocation. Our data showed an association between EBP type and treatment completion. However, it cannot be discerned whether this finding is attributable to differences between the two treatments or an artifact of selection bias since patients are not randomized into a treatment condition in clinical practice.

Despite attrition rates comparable with in-person EBP delivery, VTH resolves many major deterrents (e.g., lack of timely access, discomfort with VHA facilities) that dissuade Veterans with PTSD from initiating EBPs (39). Put differently, VTH is reaching a broader audience of patients who might otherwise not seek in-person care. Providers can leverage the flexibility of VTH as a resolution for temporary and persistent logistical barriers to in-person care. Patients feel as though their individual needs are at the forefront of clinical care, which, in turn, promotes engagement. This may partly explain our finding showing a possible dose advantage for VTH. Dose advantage refers to the tendency for patients receiving treatment remotely to complete more sessions than those receiving in-person care (19). Veterans in our study completed at least six EBP for PTSD sessions, whereas dropout among Veterans receiving these EBPs in-person typically occurs before session 5 (8, 19, 40, 41). Offering VTH as-needed may, thus, promote increased retention in EBPs.

A relative strength of this study is that it provides a longitudinal view of VTH utilization for EBPs in a single setting from 2016 to 2018. Nevertheless, our findings should be interpreted in light of study limitations. First, our data were derived from a single VHA PTSD clinic within a large VHA medical center and may not generalize to other VHA settings, such as smaller or more rural facilities. Of note, our sample size was relatively small, but mirrors the slow growth of VTH use within VHA nationally (13). Second, data were extracted from medical records that are limited by the validity of Veterans' reports, accuracy of clinicians' treatment records, and data integrity of the chart review. Finally, there was no comparison group of Veterans with PTSD receiving EBPs in-person in this specific clinic. Having the comparison group might have better elucidated differences between Veterans who used VTH and those who did not.

## General Conclusions and Future Directions

Home-based telemental health services, such as VTH, are revolutionizing mental healthcare delivery. VTH affords a high degree of flexibility that optimizes patient choice and eliminates major disruptions in care due to practical or logistical issues. With VTH, providers can remotely connect with a broad range of patients to deliver patient-centered, evidence-based care that might have otherwise been inaccessible. Despite these advantages, widescale adoption of VTH into clinical practice remains slow (13). More research on uptake and utilization of VTH in routine clinical practice as well as factors that impact its adoption (e.g., provider attitudes, clinic structure/workforce size) is needed to facilitate implementation efforts. Future work should also address provider concerns and increase their comfort with VTH since provider attitudes have influenced the uptake of this innovation (42).

As VTH becomes more integrated into routine clinical practice, it will be important to gain a richer understanding of issues related to patient engagement and EBP completion. Our study indicated a potential dose advantage for VTH, although attrition rates were comparable to in-person delivery of EBPs. Future studies should consider mixed method research designs to better understand factors related to EBP engagement, especially factors that are unique to VTH administration. Knowing this information will inform best practice standards for VTH-delivery of EBPs and enhance the quality of care delivered through this modality.

## Data Availability Statement

The datasets for this study will not be made publicly available because the dataset contains Veteran personal health information. Privacy laws prevent us from sharing personal health information outside the VHA. The Michael E. DeBakey Veterans Affairs Medical Center will not provide unrestricted, open public access to large scale health related datasets because of re-identification concerns and the obligation to protect Veterans' private information.

## Ethics Statement

The study protocol was reviewed and approved by the Institutional Review Board at Baylor College of Medicine and Affiliated Hospitals in addition to the Research and Development committee at the Houston Veterans Affairs Medical Center. Because this study involved a retrospective review of patient medical records, a waiver of informed consent/HIPAA authorization was required and granted. De-identified data were collected in a SPSS database securely stored behind a VHA firewall only accessible by the principal investigator and approved research staff. Identifiable information was only used to collect aggregate data from the medical records and no identifiable data was downloaded, copied, or printed.

## Author Contributions

All authors contributed to conception of the study. TF, DB, JL, and FK designed the study. DB and FK organized the database. DB completed data collection and performed statistical analyses. DB, FK, and TF wrote sections of the manuscript. All authors contributed to manuscript revision, read and approved the submitted version.

## Funding

This work was partly supported by the use of facilities and resources of the Houston VA HSR&D Center for Innovations in Quality, Effectiveness and Safety (CIn13-413), the Office of Academic Affiliations, Advanced Fellowship Program in Mental Illness Research and Treatment, and the VA South Central Mental Illness Research, Education and Clinical Center. The opinions expressed are those of the authors and not necessarily those of the Department of Veterans Affairs, the U.S. government, or Baylor College of Medicine. None of these bodies played a role in study design; in the collection, analysis and interpretation of data; in the writing of the report; or in the decision to submit the article for publication.

## Conflict of Interest

The authors declare that the research was conducted in the absence of any commercial or financial relationships that could be construed as a potential conflict of interest.
